# Nano-Encapsulated Spicule System Enhances Delivery of Wharton’s Jelly MSC Secretome and Promotes Skin Rejuvenation: Preclinical and Clinical Evaluation

**DOI:** 10.3390/ijms262010024

**Published:** 2025-10-15

**Authors:** Na Eun Lee, Ji Eun Kim, Chi Young Bang, Oh Young Bang

**Affiliations:** 1 Department of Health Sciences and Technology, Graduate School, Samsung Advanced Institute for Health Sciences & Technology (SAIHST), Sungkyunkwan University, Seoul 06351, Republic of Korea; cosmos1021@naver.com; 2 S&E bio, Inc., Seoul 06351, Republic of Korea; jieunkim@snebio.com; 3 Department of Plastic and Reconstructive Surgery, Kangwon National University, Chuncheon 24341, Gangwon-do, Republic of Korea; bangcy@naver.com; 4 Department of Neurology, Samsung Medical Center, Sungkyunkwan University School of Medicine, Seoul 06351, Republic of Korea

**Keywords:** spicules, mesenchymal stem cells, secretome, extracellular vesicles, nanoencapsulation, transdermal delivery, skin rejuvenation

## Abstract

Wharton’s Jelly-derived mesenchymal stem cell (WJ-MSC) secretome contains diverse bioactive factors with potential for skin regeneration, but its clinical efficacy is limited by poor transdermal delivery. In this study, we developed a dual-delivery system by nanoencapsulating WJ-MSC secretome and coating it onto marine sponge-derived spicules. Physicochemical characterization, in vitro assays (fibroblast and keratinocyte proliferation, keratinocyte migration, type I procollagen secretion, and antioxidant activity), and in vivo penetration studies were conducted. A single-arm clinical trial evaluated dermal absorption, pore characteristics, skin texture, wrinkles, and pigmentation following topical application. Transdermal penetration efficiency was significantly higher in the nano-coated spicule group than in the uncoated secretome control. In vitro, secretome treatment promoted fibroblast and keratinocyte activity, accelerated wound closure, and increased collagen synthesis. Clinically, a single application enhanced dermal absorption and significantly reduced pore number, while two weeks of treatment decreased wrinkles and pigmentation. Spicule-based nanoencapsulation effectively overcomes the skin barrier, enhances the regenerative activity of WJ-MSC secretome, and induces measurable clinical improvements in skin rejuvenation. This platform represents a promising cosmetic and therapeutic strategy in dermatology.

## 1. Introduction

Skin aging is a multifactorial process influenced by both intrinsic and extrinsic factors. Intrinsic contributors include cellular senescence and genetic predisposition, whereas extrinsic stressors, such as ultraviolet (UV) irradiation, oxidative stress, and environmental pollutants, accelerate degenerative changes [1]. These cumulative insults lead to extracellular matrix (ECM) degradation, loss of dermal elasticity, impaired barrier function, and visible manifestations including wrinkles and dyspigmentation [2,3,4]. Conventional cosmeceuticals and topical formulations often target single molecular pathways (e.g., antioxidants, retinoids, peptides); however, their efficacy is limited by poor penetration through the stratum corneum, which permits < 5–10% absorption of most hydrophilic or macromolecular actives [5,6,7,8]. Therefore, there remains an unmet need for innovative approaches that address the multifactorial biology of skin aging while overcoming the formidable skin barrier [9].

To overcome these limitations, regenerative strategies employing stem cells, their secretome, and extracellular vesicles (EVs) containing a wide array of bioactive molecules have been actively investigated [10,11]. Previous studies have demonstrated that mesenchymal stem cell (MSC)-derived secretome exerts potent anti-inflammatory effects, promotes collagen synthesis, and enhances wound healing, suggesting its potential as a cell-free therapy for skin rejuvenation [12,13,14]. Unlike single-pathway actives, secretome provides a multimodal repertoire capable of modulating multiple hallmarks of aging from ECM remodeling to immune regulation [14,15]. However, the therapeutic utility of secretome is intrinsically limited by its hydrophilicity and size heterogeneity, thereby limiting passive transdermal penetration [7]. Various approaches have been developed to address this obstacle, such as ablative and non-ablative laser treatments, microneedling, needle-free injectors, and formulation-based delivery systems designed to enhance skin penetration [16,17]. Although these methods can transiently disrupt or bypass the stratum corneum, their cosmetic application is often limited by invasiveness, cost, or potential adverse effects. Spicules, microscopic needle-like structures derived from marine sponges, have recently emerged as a promising transdermal delivery platform [18]. Spicules can transiently disrupt the stratum corneum thereby facilitating the delivery of therapeutic agents. Their unique structure and intrinsic biocompatibility have attracted growing attention as minimally invasive mediators for the delivery of drugs and biomolecules [19,20]. Spicules provide a minimally invasive and biocompatible alternative, enabling efficient penetration of therapeutic agents with a favorable safety profile [21,22].

Based on this rationale, we hypothesized that combining MSC secretome with spicules could overcome the skin barrier and achieve clinically meaningful anti-aging effects. In the present study, we developed nano-coated spicules using Wharton’s jelly-derived MSC (WJ-MSC) secretome and marine sponge-derived spicules via spray-coating technology (SCT) [23,24]. We then evaluated their biological efficacy and dermal absorption in preclinical studies, followed by a single-arm clinical study evaluating improvements in absorption, pore characteristics, skin texture, wrinkles, and pigmentation.

## 2. Results

### 2.1. Characterization of WJ-MSC Secretome and Secretome-Derived EVs

**Figure 1:**
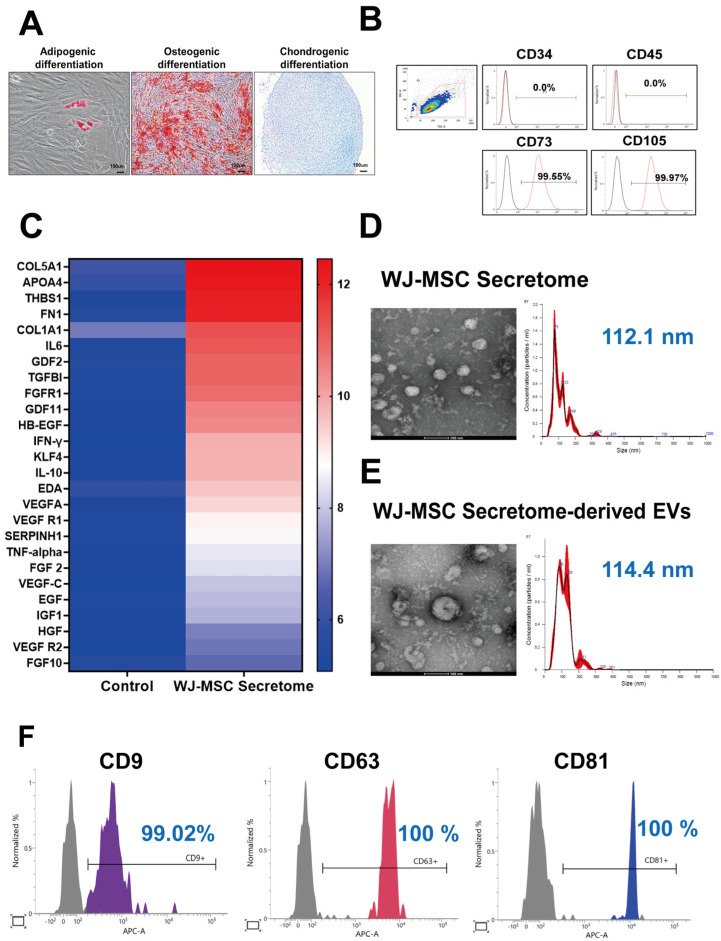
Characterization of WJ-MSCs, secretome, and extracellular vesicles (EVs). (**A**) Trilineage differentiation potential of WJ-MSCs into adipogenic, osteogenic, and chondrogenic lineages (representative images from *n* = 3 independent experiments). (**B**) Flow cytometry analysis showing positive expression of MSC markers (CD73, CD105) and negative expression of hematopoietic markers (CD34, CD45) (*n* = 3 independent runs). (**C**) Antibody array profiling of WJ-MSC secretome components. (**D**) Transmission electron microscopy (TEM) and nanoparticle tracking analysis (NTA) of WJ-MSC secretome (mean size 112.1 nm; concentration 9.15 × 10^12^ particles/mL; *n* = 2 independent isolations). (**E**) TEM and NTA of WJ-MSC secretome-derived EVs. EVs within the secretome were isolated using ExoDisc (LabSpinner, Ulsan, South Korea) (mean size 114.4 nm; concentration 8.74 × 10^10^ particles/mL; *n* = 2 independent isolations). (**F**) Surface marker expression (CD9, CD63, CD81) of secretome-derived EVs analyzed by MACSPlex (*n* = 2 biological replicates).

WJ-MSCs exhibited multipotent differentiation into adipogenic, osteogenic, and chondrogenic lineages as demonstrated by Oil Red O, Alizarin Red S, and Alcian Blue staining, respectively (Figure 1A). Flow cytometric analysis confirmed an MSC phenotype with absent hematopoietic markers (CD34, 0.0%; CD45, 0.0%) and high expression of MSC markers (CD73, 99.55%; CD105, 99.97%) (Figure 1B). Secretome profiling using a human antibody array (normalized log_2_ signal ≥ 6.0) revealed enrichment of multiple bioactive factors, including COL5A1, APOA4, FN1, COL1A1, TGF-β1, GDF11, SERPINH1, VEGF, EGF, and FGF2 (Figure 1C). Nanoparticle tracking analysis (NTA) of the crude secretome identified a predominant particle population with a mean diameter of 112.1 nm at 9.15 × 10^12^ particles/mL (Figure 1D). EVs isolated from the secretome displayed a round vesicular morphology under TEM and a mean size of 114.4 nm with a concentration of 8.74 × 10^10^ particles/mL by NTA (Figure 1E). MACSPlex profiling confirmed strong expression of EV tetraspanins CD9 (99.02%), CD63 (100%), and CD81 (100%) (Figure 1F). Collectively, these data verify the MSC identity of the source cells and demonstrate that the WJ-MSC secretome contains a cytokine-rich milieu and an abundant EV fraction bearing hallmark surface markers.

### 2.2. In Vitro Evaluation of the Skin-Rejuvenation Potential of WJ-MSC Secretome

#### 2.2.1. Cell Viability

**Figure 2:**
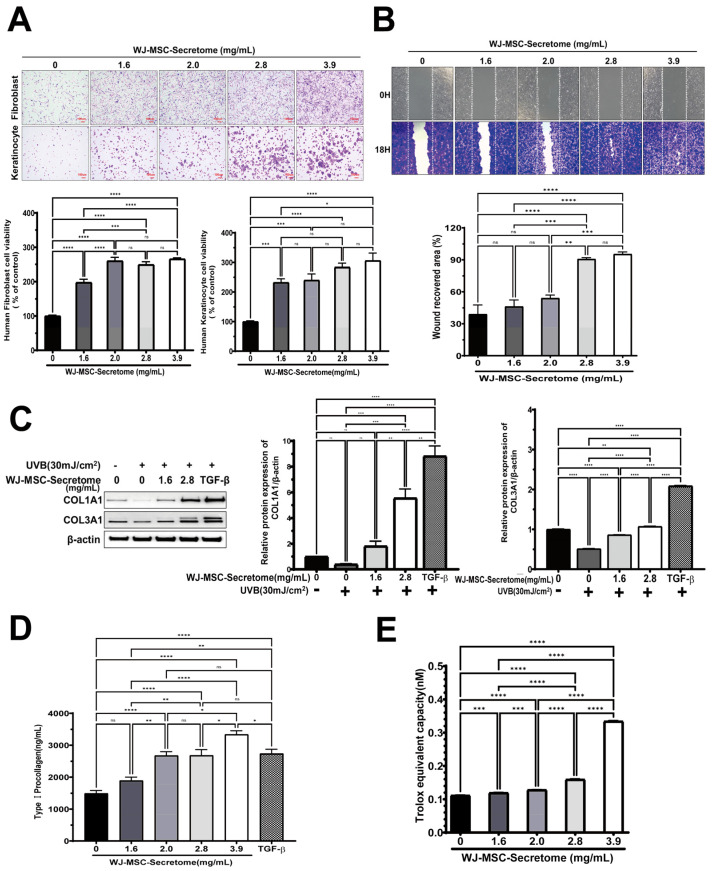
Effects of WJ-MSC secretome on skin cell function. (**A**) Proliferation of fibroblasts (HS68) and keratinocytes (HaCaT) treated with WJ-MSC secretome (0–3.9 mg/mL) for 24 h (*n* = 5 independent replicates per group). (**B**) Scratch wound healing of HaCaT cells after 18 h treatment with WJ-MSC secretome (*n* = 4 independent replicates per group). (**C**) UVB-irradiated fibroblasts (30 mJ/cm^2^) showing restored collagen (COL1A1180–200 kDa; COL3A1110/140 kDa) expression upon WJ-MSC secretome treatment. β-actin (43 kDa) was used as a loading control; uncropped blots are provided in Supplementary Figure S1. TGF-β served as a positive control (*n* = 3 independent experiments). (**D**) Type I procollagen secretion in fibroblasts following WJ-MSC secretome treatment (*n* = 5 independent replicates). (**E**) Antioxidant activity assessed by Trolox equivalent antioxidant capacity (TEAC) assay (*n* = 5 independent replicates). Data are presented as mean ± SEM. * *p* < 0.05, ** *p* < 0.01, *** *p* < 0.001, **** *p* < 0.0001.

WJ-MSC secretome significantly increased the viability of human dermal fibroblasts (HS68) and keratinocytes (HaCaT) in a concentration-dependent manner (Figure 2A). In fibroblasts, all tested concentrations (1.6-3.9 mg/mL) resulted in higher viability than the untreated control (*p* < 0.0001). In keratinocytes, viability was likewise elevated at every dose (*p* ≤ 0.001), with the greatest increase at 3.9 mg/mL. Overall, the effect became prominent at concentrations ≥ 2.0 mg/mL.

#### 2.2.2. Wound Closure

Scratch assays demonstrated dose-dependent acceleration of wound closure in keratinocytes with increasing secretome concentrations (Figure 2B). Wound recovery at 2.8 and 3.9 mg/mL was significantly greater than control (both *p* < 0.0001), while intermediate concentrations produced smaller yet significant improvements (1.6 vs. 2.8 or 3.9 mg/mL, both *p* < 0.001; 2.0 vs. 2.8 or 3.9 mg/mL, *p* = 0.001). No difference was observed between 2.8 and 3.9 mg/mL (ns, *p* = 0.965), indicating a plateau in efficacy at higher concentrations.

#### 2.2.3. Collagen Production

Western blotting demonstrated that secretome treatment restored UVB-suppressed expression of COL1A1 and COL3A1 in a concentration-dependent manner (Figure 2C). At 2.8 mg/mL, both proteins were markedly increased compared with UVB alone (COL1A1: *p* = 0.0002; COL3A1: *p* < 0.0001), reaching levels comparable to those induced by the TGF-β positive control. Consistently, ELISA revealed dose-dependent increases in type I procollagen secretion in fibroblasts (Figure 2D). Levels at 2.0, 2.8, and 3.9 mg/mL were significantly higher than control (all *p* < 0.0001), with 3.9 mg/mL exceeding both 2.0 and 2.8 mg/mL (*p* = 0.0147 and *p* = 0.0156). Notably, 3.9 mg/mL slightly exceeded the TGF-β positive control (*p* = 0.0291) (Figure 2C). For Western blot-based quantification of COL1A1/COL3A1, *n* = 3 independent biological experiments were performed. Band intensities were normalized to β-actin and analyzed by one-way ANOVA with Tukey’s post hoc. For Type I procollagen ELISA, *n* = 5 independent replicates were assayed and analyzed by one-way ANOVA followed by Tukey’s post hoc test.

#### 2.2.4. Antioxidant Capacity

Trolox-equivalent antioxidant capacity increased with secretome dose, peaking at 3.9 mg/mL (*p* < 0.0001 vs. control). Lower doses (1.6–2.8 mg/mL) produced modest increases versus control but were significantly lower than 3.9 mg/mL (all *p* < 0.0001). TEAC increased in a dose-dependent manner, indicating enhanced ROS-scavenging capacity of the WJ-MSC secretome (Figure 2E).

### 2.3. Characterization of WJ-MSC Secretome-Coated Spicules

**Figure 3:**
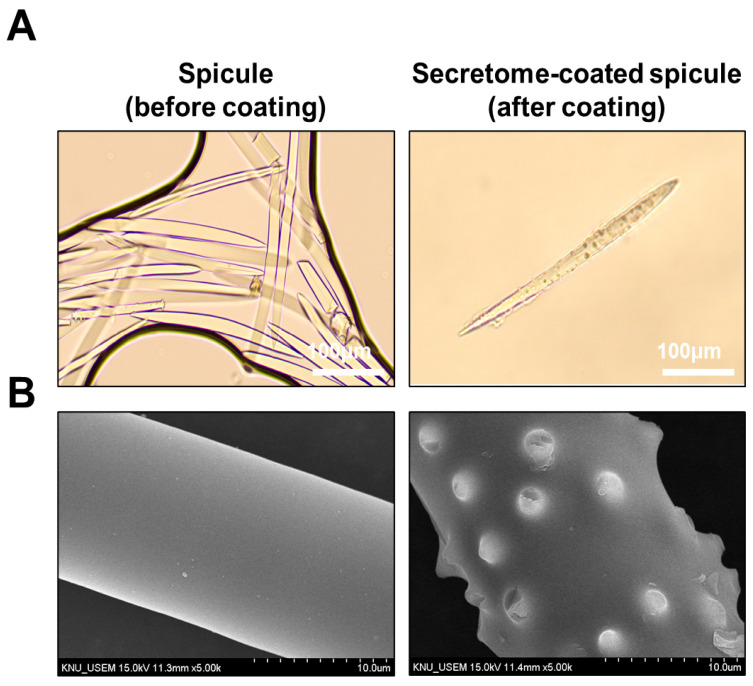
Morphological characterization of spicules with or without WJ-MSC secretome coating. (**A**) Bright-field images of uncoated (**left**) and secretome-coated (**right**) spicules (scale bar = 100 μm). (**B**) SEM images showing a smooth uncoated surface (**left**) and deposition of secretome-derived nano-deposits on coated spicules (**right**) (scale bar = 10 μm).

Bright-field microscopy revealed uncoated spicules with a smooth, needle-like morphology, whereas coated spicules displayed a peripheral coating layer along the shaft, consistent with secretome deposition (Figure 3A). Scanning electron microscopy revealed a uniformly smooth surface on uncoated spicules and a roughened surface with nanoparticulate deposits on coated ones, suggestive of secretome deposition (Figure 3B).

### 2.4. Transdermal Penetration of the WJ-MSC Secretome Using Spicules

**Figure 4:**
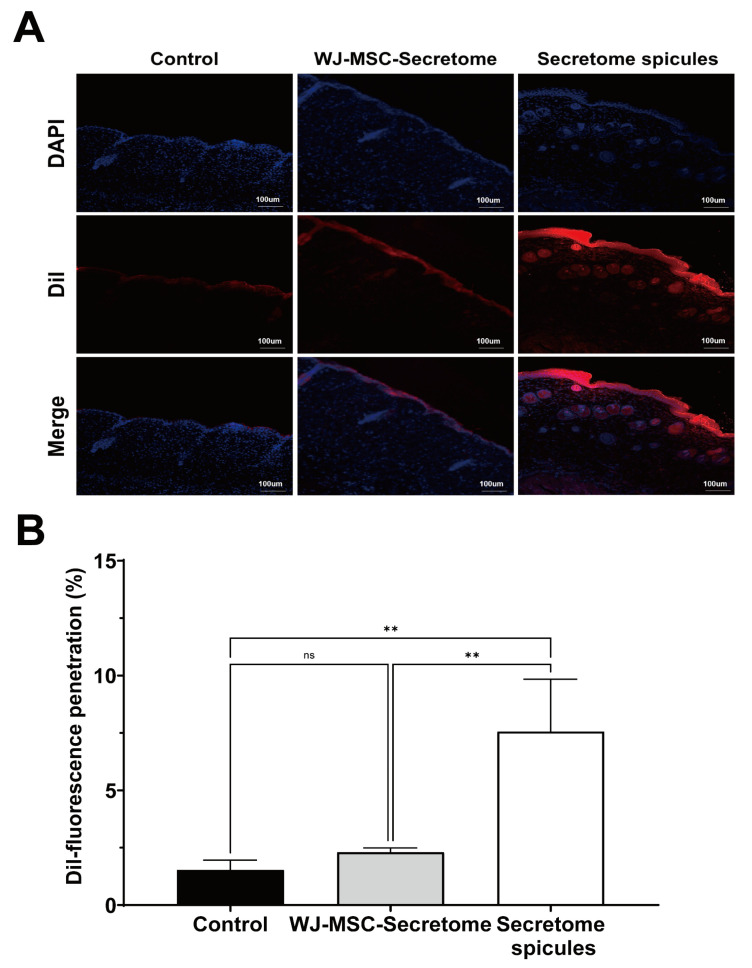
Skin penetration of secretome-coated spicules in mice. (**A**) Representative confocal images of dorsal skin sections collected 4 h after topical application of control (DiI), WJ-MSC secretome, or secretome-coated spicules. Nuclei were stained with DAPI (blue), and DiI-labeled secretome is shown in red. Scale bar = 100 μm. Dye-only controls were included to exclude false-positive DiI fluorescence signals (Supplementary Figure S2). (**B**) Quantification of DiI fluorescence penetration using ImageJ version 1.53t (*n* = 3 mice per group). Data are presented as mean ± SEM. Statistical analysis was performed by one-way ANOVA with Tukey’s multiple comparison test. ns = not significant, ** *p* < 0.01.

DiI-labeled WJ-MSC secretome was applied topically either alone or after being coated onto spicules, and fluorescence was evaluated 4 h after application (Figure 4A). In the control and secretome-alone groups, fluorescence was largely confined to the superficial epidermis. By contrast, secretome-coated spicules produced markedly deeper and more intense fluorescence extending through the epidermis into the superficial dermis. Quantitative analysis confirmed that secretome-coated spicules achieved significantly greater penetration than either control (*p* = 0.0152) or secretome alone (*p* = 0.0096), whereas the secretome-alone group did not differ from the control (ns, *p* = 0.9050) (Figure 4B). Consistently, DiI fluorescence delineated the nano-coated spicules, and histological sections confirmed spicules traversing the epidermis (Supplementary Figure S1).

### 2.5. Clinical Evaluation of WJ-MSC Secretome-Coated Spicules

#### 2.5.1. Enhanced Skin Absorption

**Figure 5:**
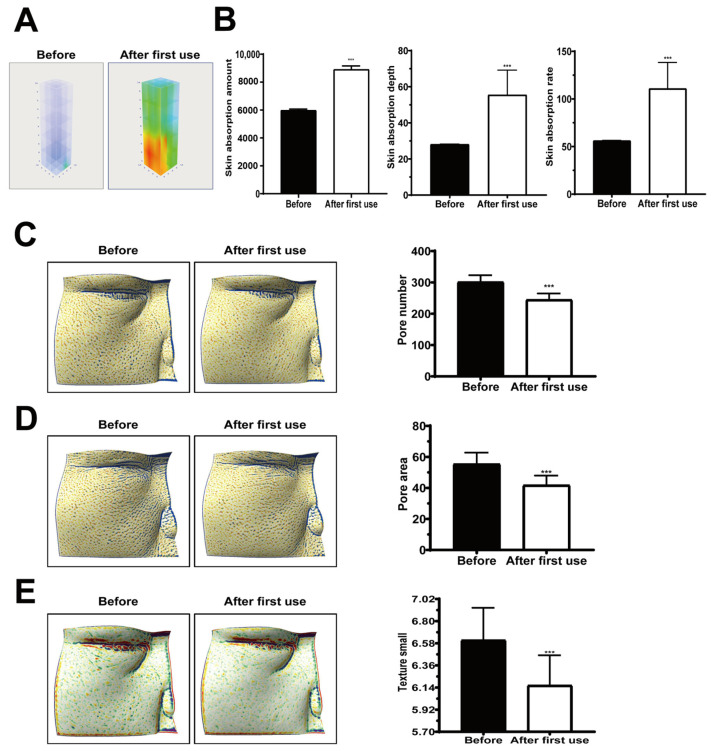
Enhanced skin absorption and skin parameter improvements after single application of nano-coated spicule essence. (**A**) Three-dimensional confocal reflectance microscopy (VivaScope, Munich, Germany) images before and after application. (**B**) Quantitative analysis showing increased skin absorption amount, depth, and rate. (**C**) Reduced pore number. (**D**) Decreased pore surface area. (**E**) Improved skin texture index. Data are presented as mean ± SEM; *** *p* < 0.001.

Three-dimensional skin imaging with quantitative readouts showed that a single application of secretome-coated spicules markedly increased the amount, depth, and rate of absorption, relative to baseline (Figure 5A,B; all *p* < 0.001).

#### 2.5.2. Pore Characteristics and Texture

Skin surface analysis showed a significant reduction in pore number and pore area immediately after the first application (Figure 5C, D; *p* < 0.001). The skin texture index also improved, indicating a smoother surface (Figure 5E; *p* < 0.001).

#### 2.5.3. Wrinkles

**Figure 6:**
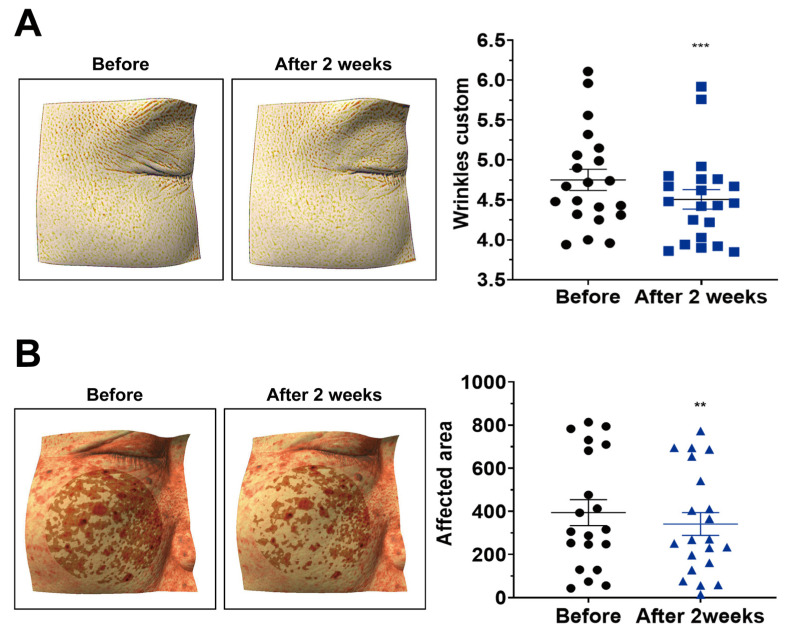
Reduction in wrinkles and pigmentation after two weeks of treatment with nano-coated spicule essence. (**A**) Wrinkle index after two weeks of treatment. (**B**) Pigmented area after two weeks of treatment. Data are presented as mean ± SEM (*n* = 21 participants). The horizontal lines in the scatter plots represent the mean ± SEM. Absolute values for wrinkle index and pigmented area are provided in Supplementary Tables S1 and S2. Statistical analyses were performed using paired *t*-test or Wilcoxon signed-rank test, as appropriate (two-tailed); ** *p* < 0.01; *** *p* < 0.001.

After two weeks of application, the wrinkle index was significantly reduced compared with baseline (Figure 6A; *p* < 0.001), indicating a visible wrinkle-reducing effect.

#### 2.5.4. Erythema

The erythema-affected area decreased after two weeks (Figure 6B; *p* < 0.01), suggesting improvement in overall skin tone.

#### 2.5.5. Adverse Events

Across all participants (*n* = 21), no adverse skin reactions were reported during the two-week period (Table 1).

## 3. Discussion

This study demonstrates that Wharton’s jelly-derived mesenchymal stem-cell (WJ-MSC) secretome enhances key cellular processes relevant to skin rejuvenation fibroblast and keratinocyte viability, keratinocyte migration, collagen production, and antioxidant capacity and that a spicule-assisted topical platform enables efficient transdermal delivery of this complex biologic cargo [18,25,26,27]. Together with quantitative in vivo penetration and exploratory clinical readouts, these findings support the feasibility of combining nano-encapsulated secretome with marine sponge-derived spicules for cosmetic dermatology applications [18,27,28]. The stratum corneum severely restricts passive diffusion of large or hydrophilic agents, consistent with classical skin-barrier principles (e.g., the 500-Dalton rule) and modern transdermal-delivery literature [29,30,31,32]. Spicules offer a minimally invasive alternative that reproducibly generates dense microchannels and improves deposition of macromolecules within the viable epidermis and superficial dermis [28,33]. This approach builds on that concept by spray-coating WJ-MSC secretome onto sponge spicules, adapting coating workflows previously developed for microneedles to achieve uniform loading [34,35]. Prior spicule-based strategies have enhanced skin delivery of peptides, siRNA, and photosensitizers often by integrating flexible liposomes, cationic carriers, or silica coatings [19,27]. To the best of current knowledge, this work demonstrates that spray-coating WJ-MSC secretome onto marine sponge spicules enables spicule-assisted transdermal delivery of a stem-cell-derived, multimolecular cargo, thereby supporting its translational potential while acknowledging related spicule-carrier approaches reported previously [33,36,37,38,39]. Mechanistically, spicules provide rapid, self-limiting barrier modulation and act as carriers of the nano-encapsulated cargo, facilitating deposition of secretome components within microperforated pathways [19,27,28,39]. This is particularly relevant to secretome biology: MSC secretome contains extracellular vesicles (EVs), growth factors, cytokines, and antioxidant enzymes that can act pleiotropically on angiogenesis, extracellular-matrix remodeling, inflammation, and oxidative stress [40,41,42]. In vitro data are consistent with this framework, demonstrating dose-dependent effects on wound closure, collagen synthesis, and antioxidant capacity (TEAC), while the in vivo mouse study confirms that secretome-coated spicules achieve significantly greater dermal penetration than either the control or secretome-alone group (see Figure 2B–E and Figure 4B) [18,19].

### 3.1. Clinical Relevance and Scope

In a short, single-arm study, a single application increased dermal-absorption metrics and improved pore-related parameters, with two weeks of twice-daily use associated with reductions in wrinkle index and pigmentation area and no adverse events reported [43,44,45]. While these early findings are encouraging for cosmetic endpoints, they should be interpreted as preliminary and hypothesis-generating rather than definitive evidence of clinical efficacy (see Figure 5 and Figure 6, Table 1) [46,47].

### 3.2. Methodological Considerations and Limitations

(1) Dosing and loading control. Spicule density, penetration depth, coating load, and application frequency were established empirically in this first-in-platform evaluation and were not exhaustively optimized [24,34]. Future design-of-experiments studies should quantify retained cargo after coating (e.g., protein content, representative growth factors by ELISA, EV number/size by NTA before vs. after coating) and correlate loading with delivery and biological response [48,49,50]. (2) Labeling specificity. Dye-only controls and stringent de-labeling (ultrafiltration ×3) were used to minimize false-positive fluorescence; nonetheless, lipophilic dyes (e.g., DiI) can form aggregates, transfer between membranes, or label non-EV components [51,52,53]. Thus, DiI fluorescence should be viewed as supportive evidence of delivery, not proof of intact EV/cargo translocation [54,55]. Orthogonal approaches protein/EV-specific labeling, detergent controls, or genetic reporters are planned to validate cargo integrity in future work [53,56]. (3) Secretome heterogeneity. Although secretome from three donors was used and independent experiments were reported, inter-donor variability in composition and potency remains a potential confounder and should be addressed with larger donor panels and batch-release assays (e.g., predefined protein/EV markers) [57,58,59]. (4) Clinical design. The clinical component was small, open-label, and single-arm, with short follow-up [60,61]. Placebo effects, regression to the mean, and site-selection bias cannot be excluded [62,63]. Blinded, randomized, vehicle-controlled trials with standardized imaging, objective biophysical readouts, and patient-reported outcomes are required to establish effect sizes and durability [64]. Head-to-head comparisons with microneedling or fractional lasers would also benchmark spicules against reference procedures [65]. (5) Safety and tolerability. Only short-term tolerability was assessed [66]. Spicule-related irritation appeared transient and aligned with prior reports, but longer-term cosmetovigilance and rare-event surveillance are necessary, particularly with repeated use and across diverse skin types [18].

### 3.3. Future Directions

Planned efforts include: (i) defining optimal dosing regimens (spicule density, coating load, frequency) using quantitative loading and delivery analytics [34]; (ii) performing orthogonal cargo-tracking and proteomic/pathway analyses in human skin to link delivery with mechanism [67]; (iii) extending applications to wound healing, scar remodeling, and inflammatory dermatoses where multimodal secretome actions may be advantageous [12,43]; and (iv) conducting powered, randomized, controlled trials with longer follow-up to determine durability and safety profiles. Collectively, the data support a pragmatic view: spicules are a scalable, minimally invasive facilitator of transdermal delivery, and WJ-MSC secretome is a biologically rich, multimolecular cargo whose pleiotropic actions may be particularly well matched to the multifactorial biology of skin aging [18,22,43]. The present study provides an initial clinical and mechanistic foothold for this combined strategy while delineating the critical experiments needed for rigorous validation and translation.

## 4. Materials and Methods

### 4.1. WJ-MSC Master Cell Banking and Secretome Preparation

Umbilical cords from three independent healthy donors (<40 years old) were collected with informed consent under IRB approval (Samsung Medical Center, IRB No. 2016-07-102-037, approved on 9 August 2022). Donor eligibility was confirmed by serological and PCR screening for HBV, HCV, HIV, HTLV-1/2, CMV, EBV, and syphilis, with all results negative. Wharton’s jelly was dissected, digested with collagenase type I (Sigma-Aldrich, St. Louis, MO, USA), and cultured in α-MEM supplemented with 15% FBS (Gibco, Thermo Fisher Scientific, Waltham, MA, USA). α-MEM with nucleosides (Gibco, Cat. No. 12571-063), formulated with L-glutamine (2.0 mM; 292 mg/L), D-glucose (1.0 g/L; 5.56 mM), sodium pyruvate (1.0 mM; 110 mg/L), and ribo- and deoxyribonucleosides; the formulation contains Earle’s salts and ascorbic acid and does not contain phenol red. Adherent MSCs were expanded to passage 4, cryopreserved, and stored in vapor-phase liquid nitrogen (<−150 °C). Quality control included sterility, endotoxin, viability, Mycoplasma, and viral safety testing. Flow cytometry confirmed >90% expression of CD44, CD73, CD90, CD105, and CD166, and <1% expression of hematopoietic markers. For secretome preparation, passage 6 WJ-MSCs from each donor were seeded at 3 × 10^3^ cells/cm^2^, cultured for 24 h, and maintained in serum-free medium for an additional 48 h. Conditioned medium from the three donors was processed separately, centrifuged, filtered through a 0.22 μm membrane (Sartorius, Göttingen, Germany), and stored at −80 °C until use. Experimental replicates were performed using secretome preparations from these three donors to account for inter-donor variability.

### 4.2. WJ-MSC Secretome Characterization

The composition of the WJ-MSC secretome was analyzed using a human antibody array (RayBio^®^ Human Antibody Array, RayBiotech, Norcross, GA, USA), which was performed by Ebiogen Inc. (Seoul, Republic of Korea). Antibody array profiling was performed once on pooled conditioned media from three independent donors (single-array exploratory screen), with identically processed unconditioned medium as the control; samples were adjusted to the manufacturer’s recommended input, and signals were background-corrected, normalized to internal negative controls, and expressed as fold-change relative to the processed control. EVs present in the secretome were further characterized by nanoparticle tracking analysis (NTA; NanoSight NS300, Malvern Instruments, Worcestershire, UK) and transmission electron microscopy (TEM; Krios G4, Thermo Fisher Scientific, Waltham, MA, USA). EVs within the secretome were isolated using ExoDisc system (LabSpinner, Ulsan, Republic of Korea), a rapid nanofiltration-based centrifugal microfluidic system for EV isolation. EV surface markers were evaluated using a MACSPlex Exosome Kit (human, Miltenyi Biotec, Bergisch Gladbach, Germany) according to the manufacturer’s protocol.

### 4.3. Trilineage Differentiation

The multipotency of MSCs was confirmed by adipogenic, osteogenic, and chondrogenic differentiation assays. Adipogenesis was visualized by Oil Red O staining, osteogenesis by Alizarin Red S staining, and chondrogenesis by Alcian Blue staining (Sigma-Aldrich, St. Louis, MO, USA).

### 4.4. Flow Cytometry

MSCs at passage 6 were stained with fluorochrome-conjugated antibodies against positive markers CD73-PE, CD105-PE, and negative markers CD34-FITC, and CD45-FITC (all from BD Biosciences, Franklin Lakes, NJ, USA). Isotype controls were included. Data were acquired and analyzed using a FACSVerse™ flow cytometer (BD Biosciences, Franklin Lakes, NJ, USA).

### 4.5. Skin Cell Culture

Human keratinocytes (HaCaT; AddexBio, San Diego, CA, USA; T0020001) and dermal fibroblasts (HS68; ATCC, Manassas, VA, USA; CRL-1635) were cultured in Dulbecco’s Modified Eagle Medium (DMEM; Gibco, Thermo Fisher Scientific, Waltham, MA, USA), supplemented with 10% fetal bovine serum (FBS; Gibco) and 1% penicillin–streptomycin (Gibco). supplemented with 10% fetal bovine serum (FBS; Gibco) and 1% penicillin–streptomycin (Gibco). Cells were maintained at 37 °C in a humidified incubator with 5% CO_2_.

### 4.6. Cell Proliferation and Viability

HS68 fibroblasts and HaCaT keratinocytes were seeded in 96-well plates at 3 × 10^3^ cells/well and treated for 24 h with WJ-MSC secretome at 0, 1.6, 2.0, 2.8, or 3.9 mg/mL (protein-equivalent). The 0 mg/mL (medium-only) condition served as the control. Cell viability was assessed using the Cell Counting Kit-8 (CCK-8; Dojindo Molecular Technologies, Rockville, MD, USA), and absorbance was measured at 450 nm using a Synergy HTX microplate reader (BioTek Instruments, Winooski, VT, USA). For qualitative assessment, HS68 and HaCaT cells were seeded in 6-well plates at 6 × 10^5^ cells/well, treated with WJ-MSC secretome for 24 h, fixed with 4% paraformaldehyde, and stained with 0.5% crystal violet (Sigma-Aldrich).

### 4.7. Scratch Wound Healing Assay

HaCaT keratinocytes were seeded in 24-well plates, grown to confluence, and scratched using a sterile pipette tip. Cells were treated with WJ-MSC secretome (0–3.9 mg/mL) and incubated for 18 h. Images were captured at 0 h and 18 h using an inverted microscope (CKX53; Olympus, Tokyo, Japan), and wound closure was quantified with ImageJ software (NIH, Bethesda, MD, USA).

### 4.8. Collagen and Antioxidant Assays

Fibroblasts were irradiated with UVB (30 mJ/cm^2^) and subsequently treated with WJ-MSC secretome. UVB irradiation (30 mJ/cm^2^) was delivered using a PCL-3000 UVB system (Boteck, Korea), with the plate positioned approximately 15 cm from the light source. Irradiance at the well surface was measured using the device’s integrated radiometer, and the exposure time required to deliver 30 mJ/cm^2^ was approximately 10 s. During irradiation, the culture medium was replaced with PBS, and the plates were uncovered (lids removed) to avoid UV attenuation; cells were immediately returned to complete medium thereafter. Expression of COL1A1 and COL3A1 was analyzed by Western blotting using specific antibodies (1:1000; Cell Signaling Technology, Danvers, MA, USA). Type I procollagen secretion was quantified using an ELISA kit (MK101; Takara Bio Inc., Shiga, Japan), with TGF-β (10 ng/mL; PeproTech, Rocky Hill, NJ, USA) as a positive control. Antioxidant activity was measured using the Trolox equivalent antioxidant capacity (TEAC) assay kit (Sigma-Aldrich) according to the manufacturer’s protocol.

### 4.9. Nano-Coated Spicules

Spicules were isolated from marine sponges (*Haliclona* sp.), purified, and air-dried. Nano-coating was performed using Spray Coating Technology (SCT), in which WJ-MSC secretome was evenly deposited onto the surface of the spicules to form a bioactive nanoscale layer. The coating procedure was carried out by Arahyeon Co., Ltd. (Anyang, Korea) under controlled laboratory conditions. The coated spicules were lyophilized and stored in a desiccator until formulation into the final essence product. Marine sponge-derived spicules were sterilized by dry heat and resuspended in sterile PBS prior to coating. For each batch, approximately 2.75 × 10^5^ spicules were combined with WJ-MSC secretome (200 µg total protein, protein-equivalent) and coated using a spray-coating system (Spray Coating Technology; Arahyeon). The secretome suspension was atomized onto the rotating spicules at 150 rpm to ensure uniform coverage, followed by air-drying at room temperature for 1 h under aseptic conditions. The coated spicules were stored in sterile, airtight containers at ambient temperature until further use.

### 4.10. In Vivo Skin Penetration Assay

The in vivo skin penetration of WJ-MSC secretome-coated spicules was evaluated using male C57BL/6 mice (6 months old). The coated spicules were labeled with 1,1′-dioctadecyl-3,3,3′,3′-tetramethylindocarbocyanine perchlorate (DiI; Thermo Fisher Scientific, Waltham, MA, USA) at a final concentration of 5 µM in PBS for 20 min at room temperature in the dark. After labeling, the DiI-stained secretome was purified by sequential ultrafiltration (100 kDa MWCO) and phosphate-buffered saline (PBS) washing steps (×3) to completely remove any unbound dye, and then gently resuspended. Following depilation of the dorsal skin 24-48 h prior to treatment, 50 μL of the DiI-labeled preparation (2.75 × 10^5^ coated spicules containing ~200 μg protein-equivalent secretome) was topically applied. Dye-only controls (spicules without secretome coating) were processed in parallel to verify labeling specificity. After 4 h, the treated skin tissues were harvested, cryosectioned, and examined using confocal laser scanning microscopy (LSM 800; Carl Zeiss, Oberkochen, Germany). Fluorescence intensity was quantified using ImageJ software (version 1.53t, NIH, Bethesda, MD, USA).

### 4.11. Formulation of Spicule-Based Essence

The spicule-based essence was prepared using a two-phase oil-in-water (O/W) emulsification process. The aqueous phase contained the emulsifier (polyglyceryl-10 stearate, 2% *w*/*w*) and humectant (glycerin, 5% *w*/*w*) dissolved in purified water, while the oil phase consisted of caprylic/capric triglyceride (6% *w*/*w*) and squalane (3% *w*/*w*). Both phases were heated separately to 80 °C; the aqueous phase was homogenized at 6000 rpm for 10 min, and the two phases were then combined under continuous stirring to form a uniform emulsion. The mixture was cooled to below 40 °C, at which point nano-coated spicules (2.75 × 10^5^ per preparation, coated with WJ-MSC secretome) were incorporated under aseptic conditions. The final formulation was filled into sterile air-tight airless containers and stored at ambient temperature until use.

### 4.12. Study Design and Participants

This single-arm, open-label clinical study was conducted in 21 healthy adult female participants aged 34–66 years. The study protocol was approved by the Institutional Review Board of the Korea Institute of Dermatological Sciences (IRB Nos. KIDSIRB-2024-1116, KIDSIRB-2024-1105, and KIDSIRB-2024-1124, all approved on 21 August 2024) and performed in accordance with the Declaration of Helsinki. Inclusion criteria included participants with healthy skin at the treatment site, exhibiting mild-to-moderate facial wrinkles, enlarged pores, or pigmentation, who provided written informed consent and agreed to refrain from using other skincare products or undergoing medical procedures during the study.

Exclusion criteria: Pregnancy or lactation; presence of active dermatologic disease (e.g., eczema, psoriasis, infection, or wounds at the application site); prior laser, filler, or chemical peeling procedures within 3–6 months; use of topical retinoids or corticosteroids within 4 weeks; known hypersensitivity to cosmetic ingredients; systemic illness or medication that could interfere with wound healing or skin evaluation. All participants were assessed by trained clinical evaluators at baseline, immediately after application, and after 2 weeks of continuous use.

### 4.13. Application Protocol

Participants were instructed to apply the WJ-MSC secretome nano-coated spicule-based essence twice daily (once in the morning and once in the evening) immediately after facial cleansing. The designated treatment area was assigned by clinical staff. Product application was continued for two weeks. Clinical evaluations were performed at three time points: baseline (before the first application), 1 h after the initial application, and after 2 weeks of continuous use.

### 4.14. Evaluation of Skin Absorption

Three-dimensional Confocal reflectance microscopy (VivaScope, Munich, Germany), a non-invasive technique to quantify molecular penetration dynamics in skin, was used to evaluate dermal absorption parameters, including absorption rate (% signal increase), penetration depth (μm), and absorption speed (time to peak signal).

### 4.15. Assessment of Pore Count and Area

Pore-related skin parameters were assessed using the ANTERA 3D imaging system (Miravex, Dublin, Ireland). All measurements were performed by the same trained evaluator to ensure consistency, targeting the right cheek of each participant. To enhance reproducibility, each post-treatment image was aligned with the corresponding baseline image using the overlay function of the ANTERA CS analysis software (version 4.6.0, Miravex, Dublin, Ireland). Pore count was quantified using the “Pores Small” filter, with the measurement variable “Count” representing the number of visible pores. Results were expressed in units of “ea” (each). In parallel, pore area was assessed using the same filter, and the variable “Affected Area” was used to quantify the total surface area occupied by pores, expressed in square millimeters (mm^2^).

### 4.16. Evaluation of Skin Texture, Fine Wrinkles, and Pigmentation

Skin texture, fine wrinkles, and pigmentation were evaluated using the ANTERA 3D imaging system (Miravex, Dublin, Ireland). All measurements were consistently performed by the same examiner on the right cheek of each participant. To ensure reproducibility, each post-treatment image was aligned with its corresponding baseline image using the overlay feature of the ANTERA CS analysis software. Skin texture improvement was assessed using the “Texture Small” filter, with surface roughness quantified by the mean Ra value. Fine wrinkles were evaluated using the “Indentation Index,” with wrinkle severity expressed through the Wrinkles Custom parameter. Pigmentation changes were analyzed using the “Melanin Hyper concentration” filter, and the total pigmented area was quantified by the Affected Area value (unit: mm^2^). A decrease in any of these parameters compared to baseline was interpreted as a positive treatment effect.

### 4.17. Assessment of Adverse Events

All participants underwent patch testing in accordance with ICDRG (International Contact Dermatitis Research Group) criteria. Adverse reactions were recorded using a standardized dermatologic assessment scale (see Table 2). Compliance and user satisfaction were also evaluated at the end of the study.

### 4.18. Statistical Analysis

In vitro assays and in vivo penetration studies were analyzed using ordinary one-way ANOVA. Clinical trial data were analyzed with SPSS software (version 17.0 for Windows; IBM Corp., Armonk, NY, USA). Normality was assessed using the Shapiro–Wilk test. Depending on data distribution, paired or independent t-tests were used for two-group comparisons, while the Wilcoxon signed-rank or Mann–Whitney U test was used for non-parametric data. For outcomes involving three or more time points, repeated measures ANOVA was performed. A *p*-value < 0.05 was considered statistically significant throughout.

## 5. Conclusions

In conclusion, we developed a third-generation regenerative cosmeceutical platform by combining WJ-MSC secretome with nano-coated sponge spicules, effectively overcoming the skin barrier and yielding clinically meaningful improvements in pore characteristics, skin texture, wrinkles, and pigmentation. Future perspectives include: (i) defining optimal dosing regimens for both spicules and secretome, (ii) expanding applications beyond anti-aging to encompass wound healing, scar remodeling, and inflammatory skin disorders, and (iii) integrating this platform with personalized dermatology approaches such as biomarker-guided treatment. Together, these findings position secretome-spicule technology as a minimally invasive, biocompatible, and versatile strategy that could reshape the landscape of regenerative dermatology.

## Figures and Tables

**Table 1:** Adverse skin reactions reported by subjects.

Adverse Reaction	After First Use/ After 2 Weeks	Adverse Reaction	After First Use/ After 2 Weeks
1. Edema (Swelling)	0	5. Spontaneous pain	0
2. Erythema (Redness)	0	6. Burning Sensation	0
3. Scaling (Flaking)	0	7. Tightness	0
4. Itching (Pruritus)	0	8. Stinging sensation	0

**Table 2:** Criteria for human skin patch test according to the International Contact Dermatitis Research Group (ICDRG).

Symbol	Score	Result
-	0	Negative
±	0.5	Doubtful or slight reaction with erythema
+	1	Erythema + induration
++	2	Erythema + induration + vesicles
+++	3	Erythema + induration + bullae

## Data Availability

The original contributions presented in this study are included in the article/Supplementary Material. Further inquiries can be directed to the corresponding author.

## Supplementary Materials

The following supporting information can be downloaded at: https://www.mdpi.com/article/10.3390/ijms262010024/s1.

## Abbreviations

The following abbreviations are used in this manuscript:

MSC	Mesenchymal stem cells
WJ-MSC	Wharton’s Jelly-derived mesenchymal stem cell
CM	Conditioned medium
EV	Extracellular vesicle
HS68	Human foreskin dermal fibroblast cell line
HaCaT	Human skin keratinocyte cell line
TGF-β	Transforming growth factor-beta
SCT	Spray Coating Technology
