# Supplementary material for: Nano-Encapsulated Spicule System Enhances Delivery of Wharton’s Jelly MSC Secretome and Promotes Skin Rejuvenation: Preclinical and Clinical Evaluation
Source: Int J Mol Sci. 2025 Oct 15;26(20):10024. doi: 10.3390/ijms262010024 (PMC12564063; doi:10.3390/ijms262010024)
Supplement: Supplementary file 1 [file ijms-26-10024-s001.zip › Supplementary Table 1.pdf]

Supplementary Table 1. pigmentation area

No.	Before	After 2 Weeks
1	476.539	403.183
2	287.121	233.377
3	412.696	410.628
4	813.171	694.237
5	247.503	230.029
6	127.698	75.792
7	252.879	250.797
8	55.473	14.168
9	730.82	772.397
10	74.213	55.595
11	305.562	268.285
12	681.635	654.579
13	782.781	693.802
14	129.332	128.011
15	315.674	196.767
16	42.816	58.29
17	794.186	540.64
18	709.493	686.929
19	246.483	161.341
20	313.755	270.885
21	393.153	365.389
AVE	390.142	341.196
SD	261.313	243.077
