# Supplementary material for: Nano-Encapsulated Spicule System Enhances Delivery of Wharton’s Jelly MSC Secretome and Promotes Skin Rejuvenation: Preclinical and Clinical Evaluation
Source: Int J Mol Sci. 2025 Oct 15;26(20):10024. doi: 10.3390/ijms262010024 (PMC12564063; doi:10.3390/ijms262010024)
Supplement: Supplementary file 1 [file ijms-26-10024-s001.zip › Supplementary Table 2.pdf]

Supplementary Table 2. Wrinkle index

No.	Before	After 2 Weeks
1	4	3.94
2	4.41	4.25
3	5.15	4.8
4	5.32	4.92
5	4.32	3.92
6	3.96	3.86
7	4.48	4.46
8	3.94	3.85
9	4.9	4.76
10	4.43	4.76
11	5.06	4.67
12	4.99	4.48
13	4.25	4.43
14	4.67	4.03
15	4.49	4.22
16	4.31	4.42
17	6.11	5.76
18	4.72	3.9
19	5.56	4.67
20	5.96	5.92
21	4.74	4.62
AVE	4.75	4.47
SD	0.61	0.56
