# Supplementary figures and images for: Nano-Encapsulated Spicule System Enhances Delivery of Wharton’s Jelly MSC Secretome and Promotes Skin Rejuvenation: Preclinical and Clinical Evaluation
Source: Int J Mol Sci. 2025 Oct 15;26(20):10024. doi: 10.3390/ijms262010024 (PMC12564063; doi:10.3390/ijms262010024)

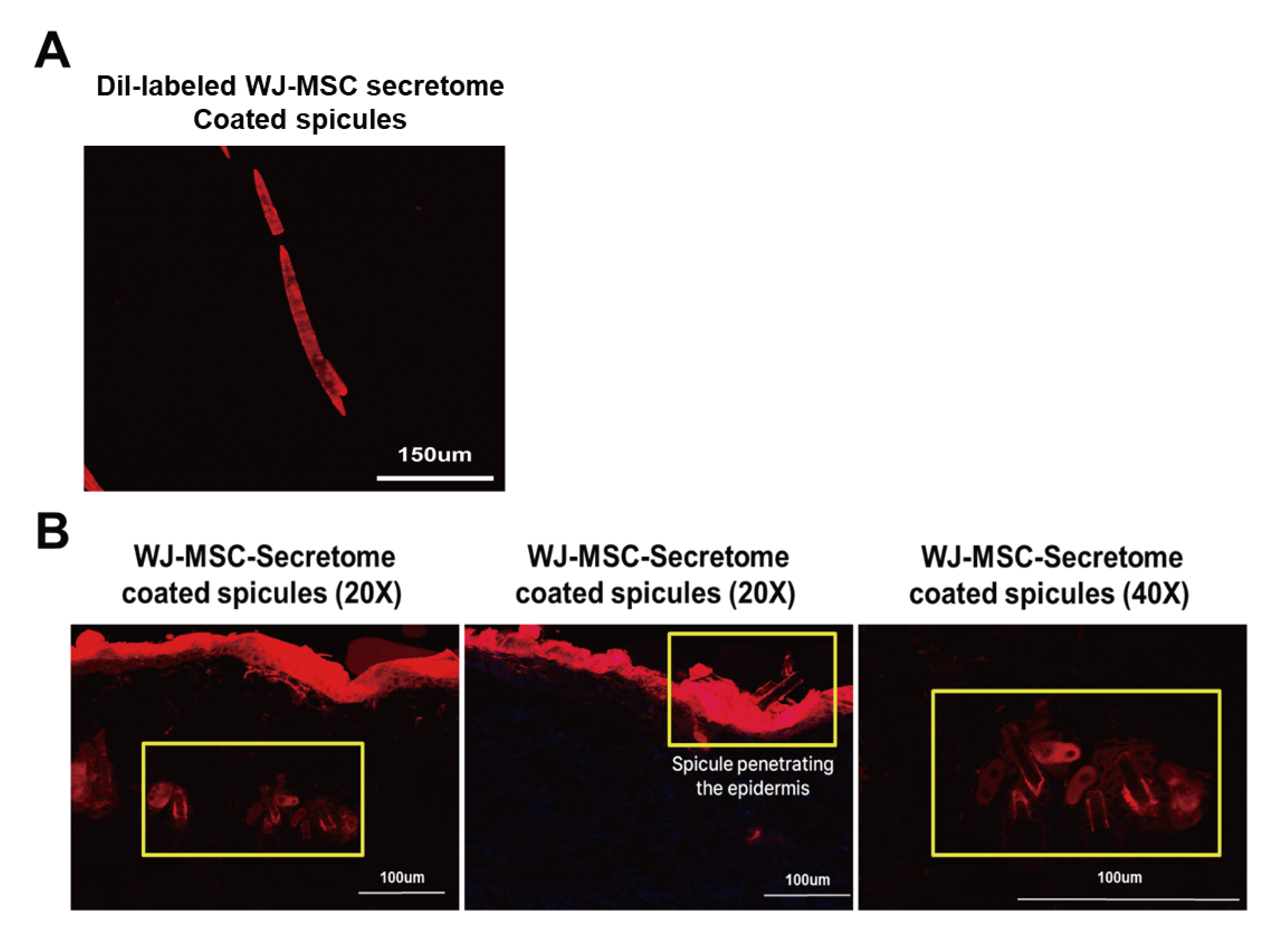

Supplement: Supplementary file 1 [file ijms-26-10024-s001.zip › Supplementary Figure 2.tif]
